# Changes in heart failure management and long-term mortality over 10 years: observational study

**DOI:** 10.1136/openhrt-2021-001888

**Published:** 2022-03-30

**Authors:** Alex Bottle, Roger Newson, Puji Faitna, Benedict Hayhoe, Martin R Cowie

**Affiliations:** 1 School of Public Health, Imperial College London, London, UK; 2 School of Cancer & Pharmaceutical Sciences, King's College London, London, UK

**Keywords:** heart failure, electronic health records, quality of health care

## Abstract

**Objectives:**

To estimate the long-term survival of two cohorts of people diagnosed with heart failure 10 years apart and to assess differences in patient characteristics, clinical guideline compliance and survival by diagnosis setting.

**Methods:**

Data for patients aged 18 and over with a new diagnosis of heart failure in the Clinical Practice Research Datalink in 2001–2002 (5966 patients in 156 practices) and 2011–2012 (12 827 patients in 331 practices). Survival rates since diagnosis were described using Kaplan-Meier plots. Compliance with national guidelines was summarised.

**Results:**

2011/2012 patients were older than those diagnosed a decade before, with lower blood pressure and cholesterol but more comorbidity and healthcare contacts. For those diagnosed in 2001/2002, the 5-year survival was 40.0% (40.2% in the 2011/2012 cohort), 10-year survival was 20.8%, and 15-year survival 11.1%. Improvement in survival between the two time periods was seen only in those diagnosed in primary care (5-year survival 46.0% vs 57.4%, compared with 33.9% and 32.6% for hospital-diagnosed patients).

Beta-blocker use rose from 24.3% to 39.1%; renin–angiotensin system blockers rose from 31.8% to 54.3% (both p<0.001). There was little change for loop diuretics and none for thiazide diuretics. For the 9963 patients with symptoms recorded by their general practitioner before diagnosis, brain natriuretic peptide (BNP) testing was low, but echocardiogram use rose from 8.3% to 19.3%, and specialist referral rose from 7.2% to 24.6% (all p<0.001).

**Conclusions:**

The 10 years saw some long-term survival gains but only modest improvement in national clinical guideline compliance, from a low baseline, despite the introduction of national initiatives.

**Key questions**

**What is already known about this subject?**

- Heart failure is a common, serious disease, often managed in primary care in the UK, but there has been little published on changes in long-term survival, prescribing and compliance with diagnosis guidelines.
- Two national quality improvement initiatives—the Quality and Outcomes Framework and the NHS Health Checks—have had some success in improving the management and short-term outcomes of chronic diseases, including heart failure. Less is known on their long-term effects.

**What does this study add?**

- In two cohorts 10 years ago, despite an older and sicker patient group in the more recent cohort, long-term all-cause mortality improved for patients diagnosed in primary care but not for those diagnosed through an emergency hospitalisation.
- Compliance with national diagnosis guidelines improved modestly from a low base.
- Prediagnosis and postdiagnosis prescribing of beta-blockers and renin–angiotensin system drugs rose over the 10 years, but loop diuretic and thiazide prescribing did not.

**How might this impact on clinical practice?**

- General practitioners need more support to improve rates of early diagnosis of heart failure.

## Introduction

Heart failure (HF) is a complex condition that affects more than 26 million people worldwide.^1^ The prevalence of HF is growing and costs US$108 billion a year globally.^2^ HF is a highly heterogeneous condition in its presentation and prognosis, so a better understanding of risk may not only improve shared decision making between patients and clinicians but also assist in identifying high-risk patients and facilitate better targeting of monitoring and potentially costly treatments.^3–5^ Most HF patients in the UK are managed in primary care by general practitioners (GPs). However, GPs face diagnostic uncertainty for patients in whom they suspect HF, which may contribute to a lack in confidence surrounding treatment selection.^6–8^ Survival has improved modestly in newly diagnosed patients since 2000 in the UK. In an analysis of a linked GP-based research database, the Clinical Practice Research Datalink (CPRD), Taylor *et al* found that the 5-year survival rate rose from 41.0% in 2000 to 48.2% in 2012, and the 10-year rate rose from 19.8% in 2000 to 26.2% in 2007.^9^ Survival was worse for those admitted to hospital around the time of diagnosis, for example, 5-year survival was 36.7% vs 51.8% for those without such an admission, and their survival rates improved less over time than those diagnosed in primary care.

Prescribing and subsequent adherence to HF medications is central to reducing HF mortality and morbidity in clinical practice.^10^ A 2005 study of six European countries including the UK found that treatment guideline adherence for ACE-inhibitors was 88% but only 55% for beta-blockers (BB), indicating variations between medication groups in addition to highlighting a need for improvement.^11^ The international QUALIFY registry, covering adult patients with reduced ejection fraction in 36 countries, found low rates of baseline prescribing and uptitration, particularly for angiotensin receptor blockers and BBs.^12^ To our knowledge, only two studies have analysed HF prescribing using CPRD data, which covers all patients with HF. Koudstaal *et al* 13 extracted HF patients from 1997 to 2010 and found renin–angiotensin system blockers (RAS), BB and mineralocorticoid receptor antagonists (MRA) were prescribed 56%, 31% and 10% of the time, respectively, among those managed in solely through primary care. Conrad *et al* 14 analysed medication prescribing in 2014 within 3 months of incident HF, and RAS, BB and MRA prescribing was 80%, 72% and 28%, respectively. Little work has directly compared prescribing trends of HF medications over time using representative data within a community setting.

Our study straddles the introduction of two key England-wide programmes aimed at reducing long-term conditions. In 2004, the Quality and Outcomes Framework (QOF), a pay-for-performance scheme in primary care, was introduced to reward GPs for better managing patients with long-term conditions, including HF. The 2009 saw the initiation of National Health Service (NHS) Health Checks, a primary care screening programme for adults aged 40–74 to detect early signs of stroke, kidney disease, heart disease, type 2 diabetes or dementia. Given this context and to better interpret the differences in mortality between the two cohorts, we also describe changes over that period in compliance with national guidelines (from NICE, the National Institute for Health and Care Excellence) on diagnosis and management in primary care and differences in drug prescription between the two time points.

## Methods

### Data

The CPRD is a database of pseudonymised electronic records from about 10% of UK general practices from 1987 to the present, considered representative of the UK population.^15^ Primary care records are linked nationally for English practices to hospital admissions (Hospital Episode Statistics, HES) and the death registry (Office for National Statistics).

### Cohort definition

A proxy HF diagnosis date was defined as the earliest mention of an HF code (see online supplemental appendix) in either primary or secondary care data. HF diagnosis dates could be in calendar years 2001–2002 or in calendar years 2011–2012. As in our previous work with CPRD,^16^ we ran sensitivity analyses using the first date of GP prescription of a loop diuretic as the HF diagnosis date, accounting for the fact that some GPs will initiate symptomatic treatment in suspected HF before formally investigating or recording a diagnosis. The results from this support the main findings and are not reported further.

10.1136/openhrt-2021-001888.supp1 Supplementary data

### Patient characteristics

A long list of patient characteristics was derived by literature review, taking into account what was recorded in CPRD. Other factors were: body mass index (BMI) in kilos per square metre, systolic blood pressure in mm Hg and Electronic Frailty Index based on polypharmacy in the previous 1 year and other deficits over the previous 5 years; population-weighted twentile of small area level socioeconomic status (Index of Multiple Deprivation, 2010); cigarette smoking category (lifelong non-smoker, ex-smoker, <10 cigarettes/day, 10–19 cigarettes/day or 20+cigarettes/day); diabetes status (none, type I or type II); ethnicity from HES (white, non-white or unknown); and a list of binary predictors. The binary factors were defined either using CPRD records in the previous 5 years, using CPRD records in the previous 1 year (before the diagnosis date), using HES records in the previous 1 year, or using combined CPRD and HES records in the previous 5 years. The CPRD binary predictors measured over the previous 5 years were: comorbidities (atrial fibrillation, arrhythmia other than atrial fibrillation, hypertension, renal diseases, myocarditis, acute myocardial infarction, congenital heart disease, coronary heart disease, chronic pulmonary disease, stroke, peripheral vascular disease); widowed or bereaved; recorded HF symptom presences (breathlessness/shortness of breath/shortness of breath on exertion), fatigue, ankle swelling). The CPRD binary factors measured over the previous year were: appointment type presences (4+min GP appointment, 4+min practice nurse appointment, home visit appointment, out of hours appointment, GP reported non-attendance, practice nurse reported non-attendance); CPRD-recorded Emergency Room visit; CPRD-recorded clinic appointment; CPRD-recorded prescriptions for a list of classes of drugs. These classes of drugs were: beta blockers, thiazide-related diuretics, loop diuretics, aldosterone antagonists, RAS drugs, glucocorticoid therapy and atypical antipsychotics. The HES binary predictors (presence indicators over the previous 1 year) were: four procedures (coronary artery bypass graft, percutaneous coronary intervention, pacemaker, implantable cardioverter defibrillators); any hospital dialysis; elective bed admission without HF primary diagnosis; emergency non-HF bed admission (1 day only); emergency non-HF bed admission (at least one night); and any hospital admissions with primary diagnosis in Clinical Classifications Software (CCS) categories (086 cataract, 122 pneumonia (except that caused by tuberculosis or sexually transmitted disease), 127 chronic obstructive pulmonary disease (COPD) and bronchiectasis, 134 other upper respiratory disease). The CCS system^17^ was devised by the US Agency for Healthcare Research and Quality as a general-purpose way of grouping ICD10 codes into homogeneous groups. Elective or emergency admission was defined using the ‘method of admission’ field in HES. The binary indicator derived from combined CPRD and HES data in the previous 5 years was living alone (online supplemental appendix).

### Statistical analysis

Crude mortality over time was described by Kaplan-Meier curves. Patient characteristics were compared between the two time points using t-tests and χ^2^ tests as appropriate.

### Patient and public involvement

Patients were not actively involved in this study.

## Results

A total of 5981 patients in 156 practices were diagnosed in 2001/2002, and 12 830 patients in 331 practices were diagnosed in 2011/12. Diagnoses could be reported by CPRD in a primary care setting (6982 patients in 342 practices) or reported by HES in a hospital setting (11 829 patients in 347 practices).

Compared with 2001/2002, patients in 2011/2012 were of similar mean age, but with a greater proportion aged 85+ (32% vs 25%) and fewer aged 65–84 (table 1). Patients were of similar body mass index (BMI) but were frailer (41% moderately or severely frail vs 21%) and more had comorbidities recorded (mean of 3 compared with 2, with 34% having 4+comorbidities in 2011/2012 compared with 13% in 2001/2002), particularly atrial fibrillation (AF), hypertension and diabetes (table 2). Fewer were current smokers in 2011/2012 (16% vs 32%). At diagnosis, the mean blood pressure (BP) was much lower in 2011/12 (9 mmHg systolic and 5 mmHg diastolic); the total and LDL cholesterol were also lower on average (all p<0.001, table 3). More patients were hospital-diagnosed in 2011/2012 (2:1, with 69% vs 31%), whereas the ratio was around 1:1 in 2001/2002. The 2011/2012 patients had more prior (non-HF) hospital admissions than 2001/2002, were more likely to have seen the GP and practice nurse, and more likely to live alone. Just over one in three had had a home visit before diagnosis in both cohorts, and around one in six had had an out-of-hours appointment with a GP before diagnosis. The recording of ethnicity in HES and BMI, smoking status and alcohol usage in CPRD improved greatly; the jump in renal disease recording from a very low base is most likely an artefact of changes in coding practice.

**Table 1:** Categorical patient characteristics at HF diagnosis in each cohort

Factor level	2001/2 cohort		2011/12 cohort	
	Frequency	Per cent	Frequency	Per cent
All patients				
Total	5981	100.0	12 830	100.0
Sex				
Male	2959	49.5	6623	51.6
Female	3022	50.5	6207	48.4
Age group in diagnosis year				
<45	57	1.0	162	1.3
45–64	716	12.0	1684	13.1
65–74	1324	22.1	2487	19.4
75–84	2383	39.8	4357	34.0
85+	1501	25.1	4140	32.3
IMD 2010 quintile				
1 (least deprived)	967	16.2	2471	19.3
2	1399	23.4	3026	23.6
3	1252	20.9	2711	21.1
4	1214	20.3	2513	19.6
5 (most deprived)	1130	18.9	2101	16.4
Unknown	19	0.3	8	0.1
HES ethnicity				
White	5037	84.2	12 251	95.5
Non-white	105	1.8	392	3.1
Unknown	839	14.0	187	1.5
Source of first HF recording				
GP consultation (CPRD)	3027	50.6	3955	30.8
Hospital admission (HES)	2954	49.4	8875	69.2
No of comorbidities				
0	904	15.1	677	5.3
1	1689	28.2	1822	14.2
2	1560	26.1	2929	22.8
3	1046	17.5	2965	23.1
4+	782	13.1	4437	34.6
Electronic Frailty Index category				
Fit	1597	26.7	1755	13.7
Mild frailty	3109	52.0	5837	45.5
Moderate frailty	1127	18.8	4296	33.5
Severe frailty	148	2.5	942	7.3
Smoking category (non, ex or current)				
Non-smoker	626	10.5	5073	39.5
Ex-smoker	422	7.1	4830	37.6
Current smoker	1893	31.7	2105	16.4
Unknown	3040	50.8	822	6.4
Alcohol drinking group				
Non drinker	232	3.9	2445	19.1
Light, moderate or unspecified	1675	28.0	4703	36.7
Heavy or alcoholic	237	4.0	730	5.7
Unknown	3837	64.2	4952	38.6
BMI group				
Underweight	95	1.6	354	2.8
Normal	910	15.2	3137	24.5
Overweight	1100	18.4	3440	26.8
Obese	836	14.0	3334	26.0
Unknown	3040	50.8	2565	20.0
Diabetes status				
No diabetes	4999	83.6	9805	76.4
Type 1 diabetes	378	6.3	732	5.7
Type 2 diabetes	604	10.1	2293	17.9

BMI, body mass index; CPRD, Clinical Practice Research Datalink; GP, general practitioner; HES, Hospital Episode Statistics; HF, heart failure; IMD, Index of Multiple Deprivation.

**Table 2:** Binary patient characteristics at HF diagnosis in each cohort

Factor	2001/2002 cohort			2011/2012 cohort		
	N present	Frequency	Per cent	N present	Frequency	Per cent
HF symptoms up to diagnosis						
Presence of: any heart failure symptom	5981	2790	46.6	12 830	7173	55.9
Presence of: breathlessness/SOB/SOBE	5981	2161	36.1	12 830	5809	45.3
Presence of: fatigue	5981	629	10.5	12 830	1685	13.1
Presence of: ankle swelling	5981	584	9.8	12 830	1716	13.4
First-symptom presence of: breathlessness	5981	1860	31.1	12 830	4934	38.5
First-symptom presence of: fatigue	5981	498	8.3	12 830	1200	9.4
First-symptom presence of: ankle swelling	5981	440	7.4	12 830	1127	8.8
Physiological indicators						
eGFR below 60 mL/min	56	23	41.1	8005	3469	43.3
Social vulnerability indicators						
Living alone	5981	342	5.7	12 830	1219	9.5
Widowed or bereaved	5981	376	6.3	12 830	761	5.9
Comorbidity components						
Comorbidity: 1 atrial fibrillation	5981	1533	25.6	12 830	5397	42.1
Comorbidity: 2 arrhythmia other than atrial fibrillation	5981	617	10.3	12 830	2346	18.3
Comorbidity: 3 diabetes	5981	979	16.4	12 830	3023	23.6
Comorbidity: 4 hypertension	5981	2528	42.3	12 830	8966	69.9
Comorbidity: 5 renal diseases	5981	363	6.1	12 830	4069	31.7
Comorbidity: 6 myocarditis	5981	82	1.4	12 830	329	2.6
Comorbidity: 7 acute myocardial infarction	5981	949	15.9	12 830	2229	17.4
Comorbidity: 8 congenital heart disease	5981	20	0.3	12 830	97	0.8
Comorbidity: 9 coronary heart disease	5981	2057	34.4	12 830	5119	39.9
Comorbidity: 10 chronic pulmonary disease	5981	1281	21.4	12 830	3440	26.8
Comorbidity: 11 stroke	5981	458	7.7	12 830	1140	8.9
Comorbidity: 12 peripheral vascular disease	5981	517	8.6	12 830	1482	11.6
NHS contacts in previous year						
CABG	5981	37	0.6	12 830	92	0.7
PTCA	5981	33	0.6	12 830	314	2.4
Pacemaker	5981	52	0.9	12 830	205	1.6
Any hospital bed admission	5981	2682	44.8	12 830	7311	57.0
Elective bed admission without HF primary diagnosis	5981	1314	22.0	12 830	3567	27.8
Emergency bed admission without HF primary diagnosis	5981	1940	32.4	12 830	5593	43.6
Emergency non-HF bed admission (1 day only)	5981	137	2.3	12 830	1045	8.1
Emergency non-HF bed admission (at least one night)	5981	1873	31.3	12 830	5173	40.3
4+min GP appointment	5981	4700	78.6	12 830	12 251	95.5
4+min practice nurse appointment	5981	2614	43.7	12 830	9243	72.0
Home visit appointment	5981	2108	35.2	12 830	4317	33.6
Out of hours appointment	5981	862	14.4	12 830	2378	18.5
Medications at baseline:						
Beta blockers (BNF chapter 2.4)	5981	1453	24.3	12 830	5013	39.1
Thiazide-related diuretics (BNF chapter 2.2.1)	5981	1158	19.4	12 830	2434	19.0
Loop diuretics (BNF chapter 2.2.2)	5981	2349	39.3	12 830	5504	42.9
Aldosterone antagonists (spironolactone or eplerenone)	5981	186	3.1	12 830	709	5.5
Renin–angiotensin system drugs (BNF chapter 2.5.5)	5981	1903	31.8	12 830	6967	54.3
Glucocorticoid therapy (BNF chapter 6.3.2)	5981	870	14.5	12 830	2464	19.2
Atypical antipsychotics (BNF chapter 4.2.1.2 or drug names)	5981	59	1.0	12 830	146	1.1

CABG, coronary artery bypass graft; eGFR, estimated glomerular filtration rate; GP, general practitioner; HF, heart failure; PTCA, percutaneous transluminal coronary angioplasty; SOB, shortness of breath; SOBE, shortness of breath on exertion.

**Table 3:** Quantitative patient characteristics at time of first recorded HF diagnosis in each cohort

Quantitative variable	2001/2002 cohort			2011/2012 cohort		
	N present	Mean	SD	N present	Mean	SD
Age in diagnosis year	5981	76.9	11.0	12 830	77.5	12.1
No of comorbidities	5981	1.9	1.4	12 830	2.9	1.6
Electronic Frailty Index	5981	0.18	0.1	12 830	0.2	0.1
BMI (kilos/square metre)	2914	27.5	5.7	10 219	28.1	6.5
Systolic blood pressure (mm Hg)	5262	145.4	22.8	12 590	133.6	19.3
Diastolic blood pressure (mm Hg)	5262	80.0	11.7	12 590	74.8	11.4
Serum cholesterol (mmol/L)	2278	5.2	1.2	11 018	4.6	1.2
High-density lipoprotein (HDL cholesterol (mmol/L)	1073	1.3	0.4	9831	1.4	0.4
Low-density lipoprotein (LDL) cholesterol (mmol/L)	751	3.1	1.0	8463	2.6	1.0
HDL/LDL cholesterol ratio	455	4.0	1.5	8818	3.5	1.2
Blood glucose (mmol/L)	2437	7.3	3.9	9540	6.5	3.0
Serum creatinine (umol/L)	3480	108.3	43.0	12 284	101.9	50.9
Serum triglycerides (mmol/L)	1371	1.8	1.2	9096	1.5	1.0
Blood urea (mmol/L)	2529	7.8	5.0	10 625	8.0	4.5
Estimated glomerular filtration rate (mL/min)	56	61.7	14.0	8005	61.7	20.5
Haemoglobin (g/dL)	3151	131.0	19.0	11 903	128.0	19.0

BMI, body mass index; HF, heart failure.

Many of these changes were evident when comparing the two cohorts by diagnosis setting (online supplemental appendix tables A2.1-2.3). For those with HF recorded first in primary care, 2011/2012 patients were more likely to be male, aged 45–64, with more comorbidities, particularly type 2 diabetes and arrhythmias. Hypertension was more commonly recorded despite lower mean BP. For hospital-diagnosed patients, the comorbidity and physiological differences were the same as for those diagnosed in primary care, with the exception of age and gender: the later cohort had an equal gender split with a higher proportion in the aged 85+ category.

The use of some HF-related medications at diagnosis rose in the 10 years, particularly for BB (from 24.3% to 39.1%, p<0.001) and RAS (from 31.8% to 54.3%, p<0.001), with little change for loop diuretics (39.3%–42.9%). Twelve months after diagnosis, BB use rose further in the 10 years (from 24.1% to 50.8%), loop diuretics were much higher than at diagnosis in both cohorts (61.5% and 55.9%), aldosterone antagonists and RAS drugs were also higher than at diagnosis in both cohorts (online supplemental appendix table A1).

Of the diagnosed patients, 9963 (2790 diagnosed in 2001/2002 and 7173 diagnosed in 2011/2012) presented with HF-specific symptoms (breathlessness, fatigue or swollen ankles) in the 5 years at or before diagnosis. We calculated Kaplan-Meier response rates for time to each of three national guideline (NICE) recommended primary care ‘responses’ (echocardiogram, brain natriuretic peptide (BNP) blood test and referral to cardiologist), starting at first presentation with symptoms in the 5 years at or before diagnosis. Table 4 shows the cumulative numbers of patients, and Kaplan-Meier response rates (allowing for censorship by exit from CPRD), in the presenting patients at 6 weeks and 6 months after presentation with symptoms. BNP tests were not performed on 2001/2002 patients (not mandated by NICE at that time), but the other two NICE-recommended responses have become more frequent, after both 6 weeks or 6 months, in 2011/2012 than they were in 2001/2002.

**Table 4:** Proportions of patients meeting each element of NICE guidelines on HF diagnosis by cohort within 6 weeks and 6 months of first presentation to GP with HF symptoms

NICE guideline element	2001/2002 cohort n (%) within 6 weeks	2001/2002 cohort n (%) within 6 months	2011/2012 cohort n (%) within 6 weeks	2011/2012 cohort n (%) within 6 months
Echocardiogram	84 (3.0%)	660 (9.2)	223 (8.3)	1362 (19.3)
BNP blood test	0	429 (6)	0	577 (8.1)
Referral to cardiologist	79 (2.9%)	870 (12.2)	194 (7.2)	1736 (24.6)

BNP, brain natriuretic peptide; GP, general practitioner; HF, heart failure; NICE, National Institute for Health and Care Excellence.

**Figure 1:**
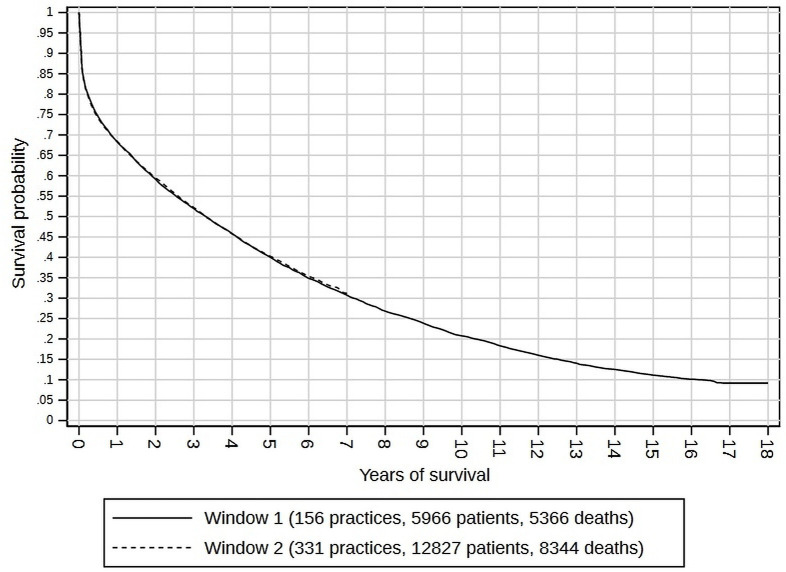
Kaplan-Meier curves for patients diagnosed in window 1 (2001–2002) and window 2 (2011–2012, with follow-up censored at 7 years).

**Figure 2:**
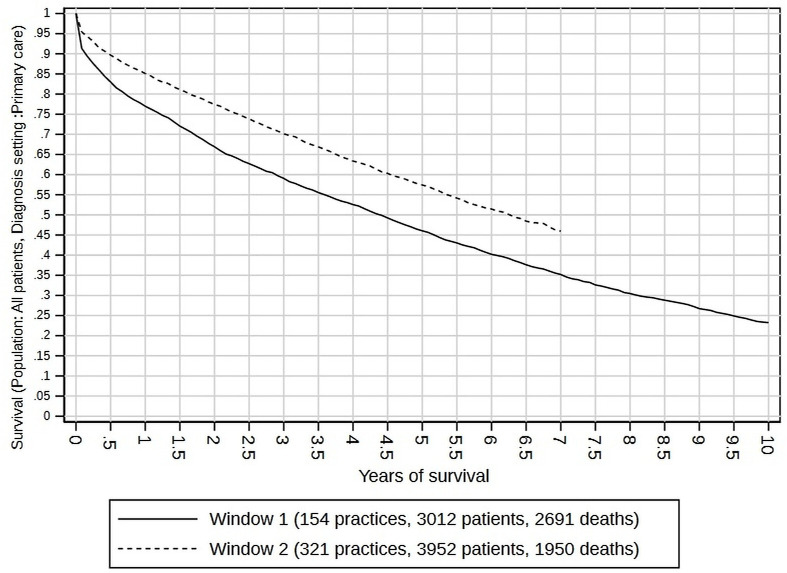
Kaplan-Meier survival curves by diagnosis cohort (window 1 is 2001–2002; window 2 is 2011–2012) for patients diagnosed in a primary care setting.

**Figure 3:**
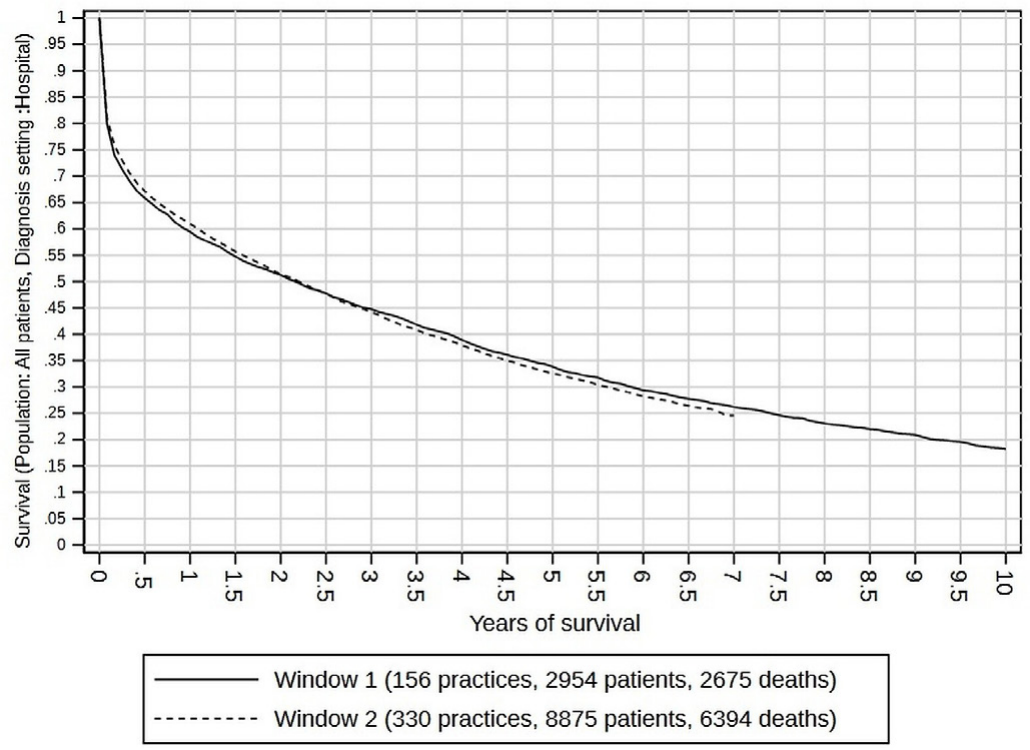
Kaplan-Meier survival curves by diagnosis cohort (window 1 is 2001/2002; window 2 is 2011/2012) for patients diagnosed in a hospital setting.

Figure 1 shows the Kaplan-Meier plots for each cohort. The 5-year survival rate was 0.400 (95% CI 0.383 to 0.417) in 2001/2002, and 0.402 (95% CI 0.391 to 0.414) in 2011/2012. For the 2011/2012 cohort, 10-year and 15-year survival rates could not be measured, as patients diagnosed in 2011–2012 did not have sufficient follow-up by 2018 (explaining the flat Kaplan-Meier curves after 7 years). However, in the 2001/2002 cohort, 10-year survival was 0.208 (95% CI 0.193 to 0.223), and 15-year survival was 0.111 (95% CI 0.101 to 0.123). Rates of survival, and their improvement, varied by diagnosis setting. Figure 2 shows the survival curves for patients diagnosed in primary care, where 5-year survival was 0.460 (95% CI 0.438 to 0.483) for the 2001/2002 cohort and had improved to 0.574 (95% CI 0.552 to 0.596) for the 2011/2012 cohort. Figure 3 shows the survival curves for patients diagnosed in hospital, where 5-year survival was 0.339 (95% CI 0.318 to 0.360) for 2001/2002 and 0326 (95% CI 0.315 to 0.337) for 2011/2012, implying poorer prospects (especially in the first 6 months) for 2001/2002 and no visible improvement for 2011/2012.

**Figure 4:**
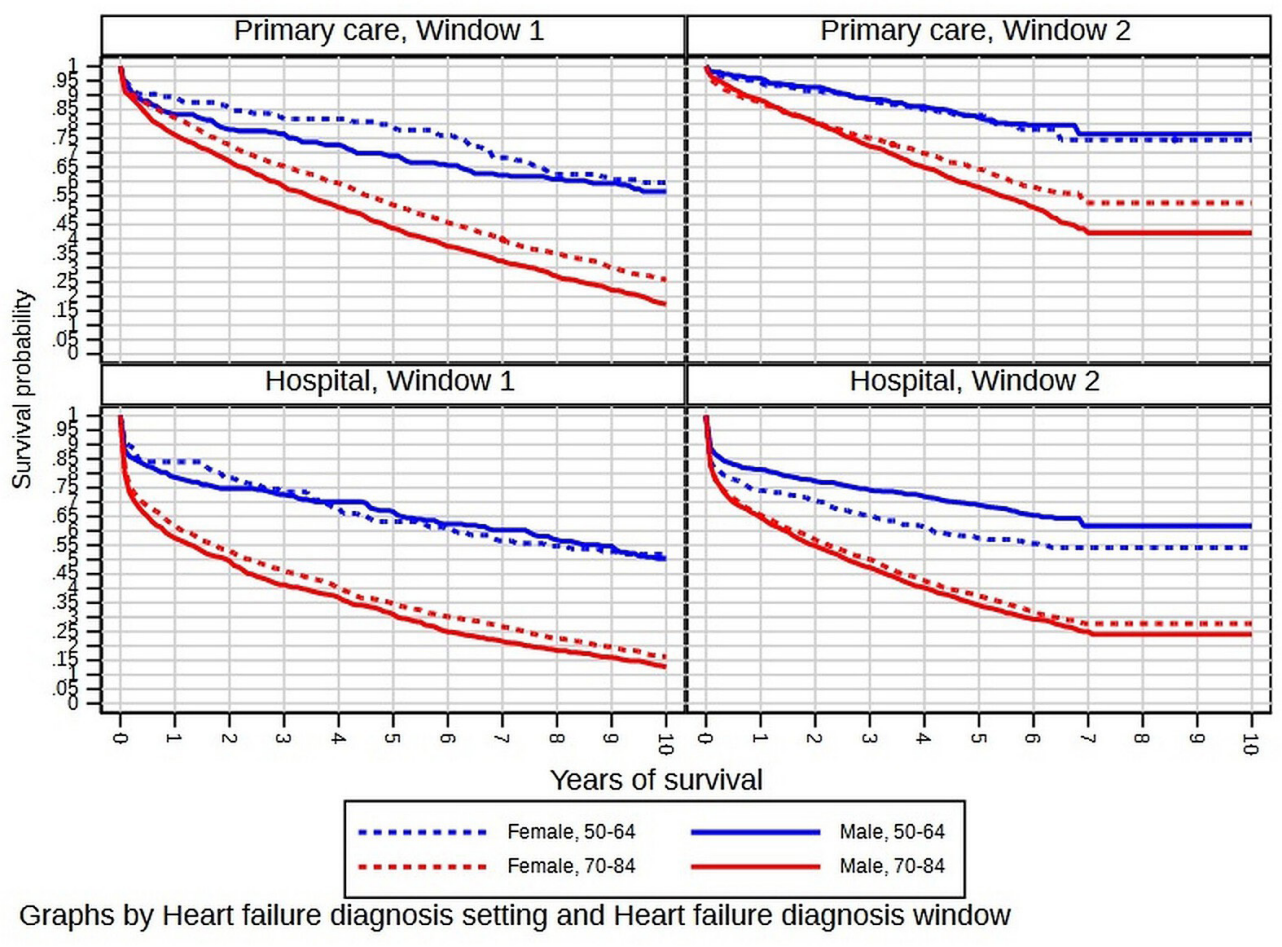
Kaplan-Meier curves for each combination of sex and age group, plotted by combination of diagnosis setting (primary care or hospital) and diagnosis window (1 or 2) in colour.

Survival varied by age and sex. Figure 4 shows the KM curves for each window, with separate curves by sex and for people aged 50–64 and for people aged 70–84. The improvement in survival over time was seen in both genders and all age groups.

## Discussion

### Summary of main findings

Long-term survival after a diagnosis of HF showed some improvement over the 10 years if the diagnosis was made in primary care but became slightly worse if the diagnosis was recorded following a hospital admission. Compliance with national (NICE) guidelines for diagnosis rose but remained low. The survival improvement was despite rises in the proportions of patients aged 85 and over in those diagnosed in hospital, and in the recorded burden of comorbidities, frailty and in living alone. The later cohort had notably lower average blood pressure and cholesterol and more prescribing of BB and RAS medications than the earlier cohort.

### Comparison with previous studies

Taylor *et al* 9 also used CPRD in the UK and found modest improvements in survival between 2000 and 2014, their 5-year figures matching ours for comparable years; they and Koudstaal *et al* 13 also found that hospital-diagnosed patients had higher mortality. Koudstaal *et al* extracted HF patients from 1997 to 2010 and found RAS, BB and MRA were prescribed 55.8%, 30.5% and 9.7% of the time, respectively, among those who managed solely through primary care. Conrad *et al* 14 analysed medication prescribing in 2014 within 3 months of incident HF, and RAS, BB and MRA prescribing was 80%, 72% and 28%, respectively. For 2011/2012, our figures were 54.3% for RAS, 39.1% for BB and 5.5% for MRA, similar to those of Koudstaal but lower than Conrad’s. The National HF Audit of those hospitalised has also shown a slow but steady improvement in the use of disease-modifying drug therapies for patients with HFrEF in UK hospitals.^18^ However, this is still likely to be suboptimal, particularly if patients are older and not under the care of a cardiologist.

Our study straddles the 2004 introduction of a pay-for-performance framework in primary care (QOF) and the 2009 initiation of a cardiovascular risk screening programme (NHS Health Checks). QOF has been shown to be associated with improvements in blood pressure and cholesterol control in patients with diabetes^19^ and in blood pressure in all patients.^20^ QOF’s financial incentives mean that at least some of the greater recording of comorbidities and frailty in our 2011/2012 cohort compared with 2001/2002 could be due to greater coding rather than higher prevalence. A difference-in-difference analysis with a 2-year follow-up of the NHS Health Checks programme found statistically significant but clinically modest impacts on modelled risk for cardiovascular disease and individual risk factors.^21^ Specifically for HF, a qualitative study from 2014 found that barriers to accurate diagnosis and effective management of HF had not changed in the 10 years since the authors’ previous study.^22^ Only a minority of HF patients have access to cardiac rehabilitation, despite its inclusion as an indicator in QOF, and there is often poor adherence to medication.^23^

That the majority of patients were diagnosed via an emergency hospitalisation in 2011/2012—in contrast to 2001/2002—agrees with our previous CPRD findings^16^ and could have several explanations. A key potential artefactual reason is coding. QOF incentivises better recording and management of long-term conditions, including HF, with some GPs delaying the coding of HF until they have a definitive diagnosis; however, others may record it earlier as a working diagnosis, so the impact on the trend is hard to judge. Other explanations could include some patients having greater difficulty in accessing GPs, a trend in patients preferring to use the ED for issues that could have been dealt with by their GP, and changing HF aetiology/phenotype. We found greater recorded comorbidity and frailty in 2011/2012 than in 2001/2002. As mentioned earlier, some of this could be due to QOF-induced detection and recording of long-term conditions, but probably not all, as the later cohort were older. If the prevalence increases are real, it could also explain the greater use of some medications in the later cohort.

### Strengths and limitations

CPRD is representative of the general UK community-dwelling population. Its linkage to HES and the death registry ensures national coverage for those outcomes. It contains the information that GPs have available to them to make decisions, however incomplete that may be. CPRD data are entered by GPs during routine consultations and not for the purpose of research, and it is recognised that coding is variable in primary care.^24^ Symptom codes are not subject to incentivisation such as QOF and are likely to show greater variation than diagnosis codes. Other CPRD limitations include missing values. Most patients had no BNP or HF type recorded. This prevents us from assessing the appropriateness of prescribing.

We used the first recorded mention of HF as the date of diagnosis. As noted above, some GPs may record a tentative diagnosis before they investigate, which is why we undertook a sensitivity analysis using the date of first loop diuretic prescription to indicate the date when the GP first suspected HF that was only confirmed later. However, other GPs may wait until the diagnosis has been confirmed through investigations or specialist referral before they record it. Like users of most data sets, we relied on the recording of HF, which in turn depends on both the diagnosis and its coding. It is known that the prevalence of cardiovascular disease reported in QOF registers falls well short of the predicted prevalence in many areas of the country, indicating underdiagnosis.^23^

Risk assessment tools need to be updated over time to account for therapy advances, but to study longer-term prognosis requires a significant time lag, as here. This inevitably means that covariates measured at baseline—when the GP made the initial management decisions—reflect clinical practice at that time, which in our study was 2001–2002 and 2011–2012. Despite this, it is notable that mortality only modestly changed during that time and, from Taylor *et al*’s 1-year survival figures,^9^ up to 2016.

We have reported mortality, but poor outcomes aside from death may affect patients with HF. We report elsewhere the risk of emergency hospitalisation,^25^ but measures such as patients’ ability to work and live independently are not captured by CPRD.

## Conclusion

These findings show that there had been some progress in reducing long-term mortality for patients diagnosed in primary care, despite increasing age and comorbidity at the time of diagnosis, at least partially explained by improvement in NICE guideline compliance for diagnosis and in disease-modifying drug prescription. Further effort is required to accelerate the time to diagnosis and to increase adherence to evidence-based guidelines.

## Data Availability

Data may be obtained from a third party and are not publicly available. The anonymised patient data that was used for this study can be accessed by contacting the Clinical Practice Research Datalink at enquiries@cprd.com. Access to these data is subject to a data sharing agreement (DSA) containing detailed terms and conditions of use following protocol approval from CPRD’s Independent Scientific Advisory Committee (ISAC).
