# Correction: *Changes in heart failure management and long-term mortality over 10 years: observational study*

**DOI:** 10.1136/openhrt-2021-001888corr1

**Published:** 2024-03-05

Bottle A, Newson R, Faitna P, *et al*. Changes in heart failure management and long-term mortality over 10 years: observational study. *Open Heart* 2022;9:e001888. doi: 10.1136/openhrt-2021-001888

This article has been corrected since it was first published. The column headings for Table 4 were incorrect, and now have been corrected to the following: 2001/2002 cohort n (%) within 6 weeks, 2001/2002 cohort n (%) within 6 months, 2011/2012 cohort n (%) within 6 weeks, 2011/2012 cohort n(%) within 6 months.

