# Supplementary material for: Changes in heart failure management and long-term mortality over 10 years: observational study
Source: Open Heart. 2022 Mar 30;9(1):e001888. doi: 10.1136/openhrt-2021-001888 (PMC8969012; doi:10.1136/openhrt-2021-001888)
Supplement: Supplementary data [file openhrt-2021-001888supp001.pdf]

Appendix

Patient characteristics

As well as age and sex, we reported: body mass index (BMI) in kilos per square metre, systolic blood pressure in mmHg and electronic frailty index (eFI) based on polypharmacy in the previous 1 year and other deficits over the previous 5 years (see Clegg et al for codes – reference is below); population-weighted twentile of small area level socio-economic status (Index of Multiple Deprivation, IMD 2010); cigarette smoking category (lifelong non-smoker, ex-smoker, <10 cigarettes/day, 10-19 cigarettes/day, or 20+ cigarettes/day); diabetes status (none, Type I or Type II); ethnicity from HES (white, non-white or unknown); and a list of binary predictors. The binary factors were defined either using CPRD records in the previous 5 years, using CPRD records in the previous 1 year (before the diagnosis date), using HES records in the previous 1 year, or using combined CPRD and HES records in the previous 5 years. The CPRD binary predictors measured over the previous 5 years were: co-morbidities (atrial fibrillation, arrhythmia other than atrial fibrillation, hypertension, renal diseases, myocarditis, acute myocardial infarction, congenital heart disease, coronary heart disease, chronic pulmonary disease, stroke, peripheral vascular disease); widowed or bereaved; recorded HF symptom presences (breathlessness/shortness of breath/shortness of breath on exertion), fatigue, ankle swelling). The CPRD binary factors measured over the previous year were: appointment type presences (4+ minute GP appointment, 4+ minute practice nurse appointment, home visit appointment, out of hours appointment, GP reported non-attendance, practice nurse reported non-attendance); CPRD-recorded Emergency Room visit; CPRD-recorded clinic appointment; CPRD-recorded prescriptions for a list of classes of drugs. These classes of drugs were: beta blockers, thiazide-related diuretics, loop diuretics, aldosterone antagonists, renin-angiotensin system (RAS) drugs, glucocorticoid therapy, and atypical antipsychotics. The HES binary predictors (presence indicators over the previous 1 year) were: four procedures (coronary artery bypass graft, percutaneous coronary intervention, pacemaker, implantable cardioverter defibrillators); any hospital dialysis; elective bed admission without HF primary diagnosis; emergency non-HF bed admission (one day only); emergency non-HF bed admission (at least one night); and any hospital admissions with primary diagnosis in AHRQ's Clinical Classification Software (CCS) categories (086 Cataract, 122 Pneumonia (except that caused by tuberculosis or sexually transmitted disease), 127 COPD and bronchiectasis, 134 Other upper respiratory disease). The CCS system was devised by the US Agency for Healthcare Research and Quality as a general-purpose way of grouping ICD10 codes into homogeneous groups. Elective or emergency admission was defined using the "method of admission" field in HES (11-13 for elective, 21-28 for emergency). The binary indicator derived from combined CPRD and HES data in the previous 5 years was living alone.

Read codes for the primary care variables were taken from our previous work (Bottle A, Kim D, Aylin P, Cowie MR, Majeed A, Hayhoe B. Routes to diagnosis of heart failure: observational study using linked data in England. Heart. 2018 Apr;104(7):600-605. doi: 10.1136/heartjnl-2017-312183).

Reference for the eFI:
Clegg A, Bates C, Young J, Ryan R, Nichols L, Teale EA, Mohammed MA, Parry J, Marshall T. Development and validation of an electronic frailty index using routine primary

care electronic health record data. Age Ageing 2016; 45(3): 353–360. https://doi.org/10.1093/ageing/afw039

Details of medications (BNF = British National Formulary)

Beta blockers (BNF Chapter 2.4); Thiazide-related diuretics (BNF Chapter 2.2.1); Loop diuretics (BNF Chapter 2.2.2); Aldosterone antagonists (spironolactone or eplerenone); Renin-angiotensin system (RAS) drugs (BNF Chapter 2.5.5); Glucocorticoid therapy (BNF Chapter 6.3.2); Atypical antipsychotics (BNF Chapter 4.2.1.2 and/or any of 10 individual drug names).

Table A1. Medications at 12 months after diagnosis

	2001/2 cohort n, % at dx	2001/2 cohort n, % 12 months since dx	2011/12 cohort n, % at dx	2011/12 cohort n, % 12 months since dx
Beta blockers	1453, 24.3%	1441, 24.1%	5013, 39.1%	6521, 50.8%
Thiazide-related diuretics	1158, 19.4%	588, 9.8%	2434, 19.0%	983, 7.7%
Loop diuretics	2349, 39.3%	3676, 61.5%	5504, 42.9%	7175, 55.9%
Aldosterone antagonists	186, 3.1%	775, 13.0%	709, 5.5%	2291, 17.9%
Renin-angiotensin system (RAS) drugs	1903, 31.8%	3272, 54.7%	6967, 54.3%	7552, 58.9%
Glucocorticoid therapy	870, 14.5%	695, 11.6%	2464, 19.2%	1791, 14.0%
Atypical antipsychotics	59, 1.0%	74, 1.2%	146, 1.1%	141, 1.1%

Table A2.1. Quantitative patient characteristics at time of first recorded HF diagnosis in each cohort, GP-diagnosed

	2001/2 cohort			2011/12 cohort		
Quantitative variable	N present	Mean	SD	N present	Mean	SD
Attained age in diagnosis year	3027	76.9	10.5	3955	75.2	12.1
Number of comorbidities	3027	1.6	1.3	3955	2.4	1.6
Electronic frailty index (eFI)	3027	0.2	0.1	3955	0.2	0.1
BMI (kilos/square metre)	1555	27.7	5.8	3281	28.5	6.3
Systolic blood pressure (mm Hg)	2709	145.5	22.5	3900	133.6	18.9
Diastolic blood pressure (mm Hg)	2709	80.2	11.5	3900	75.6	11.2
Serum cholesterol (mmol/L)	1225	5.3	1.1	3513	4.6	1.2
HDL cholesterol (mmol/L)	582	1.4	0.4	3169	1.4	0.4
LDL cholesterol (mmol/L)	404	3.1	1.0	2727	2.6	1.0
HDL/LDL cholesterol ratio	240	4.0	1.4	2856	3.5	1.2
Blood glucose (mmol/L)	1281	7.1	3.5	2946	6.2	2.5
Serum creatinine (umol/L)	1817	106.2	39.5	3817	96.2	38.9
Serum triglycerides (mmol/L)	729	1.7	1.1	2906	1.4	1.1
Blood urea (mmol/L)	1361	7.6	4.9	3247	7.4	3.5
Estimated glomerular filtration rate (mL/min)	31	62.0	12.4	2513	64.6	18.8
Haemoglobin (g/dL)	1629	13.2	1.8	3702	13.2	1.8

Table A2.2. Categorical patient characteristics at HF diagnosis in each cohort, GP-diagnosed

	2001/2 cohort		2011/12 cohort	
Factor level	Frequency	Percent	Frequency	Percent
All patients:				
Total	3027	100	3955	100
Gender:				
Male	1505	49.7	2253	57.0
Female	1522	50.3	1702	43.0
Age group in diagnosis year:				
<45	21	0.7	68	1.7
45-64	345	11.4	649	16.4
65-74	693	22.9	921	23.3
75-84	1253	41.4	1393	35.2
85+	715	23.6	924	23.4
IMD 2010 quintile:				
1 (least deprived)	509	16.8	789	19.9
2	715	23.6	945	23.9
3	670	22.1	882	22.3
4	598	19.8	726	18.4
5 (most deprived)	528	17.4	612	15.5
Unknown	7	0.2	1	0.0
HES ethnicity (white or non-white):				
White	2491	82.3	3765	95.2
Non-white	56	1.9	102	2.6
Unknown	480	15.9	88	2.2
Number of comorbidities (group):				
0	649	21.4	420	10.6
1	985	32.5	813	20.6
2	727	24.0	965	24.4
3	412	13.6	819	20.7
4+	254	8.4	938	23.7
Electronic frailty index (eFI) category:				
Fit	551	18.2	428	10.8
Mild frailty	1693	55.9	1846	46.7
Moderate frailty	695	23.0	1367	34.6
Severe frailty	88	2.9	314	7.9
Smoking category (non, ex or current):				
Non-smoker	344	11.4	1562	39.5
Ex-smoker	233	7.7	1573	39.8
Current smoker	985	32.5	613	15.5

Unknown	1465	48.4	207	5.2
Alcohol drinking group:				
Non drinker	109	3.6	683	17.3
Light, moderate or unspecified	945	31.2	1646	41.6
Heavy or alcoholic	129	4.3	261	6.6
Unknown	1844	60.9	1365	34.5
BMI group:				
Underweight	49	1.6	89	2.3
Normal	468	15.5	909	23.0
Overweight	592	19.6	1181	29.9
Obese	460	15.2	1116	28.2
Unknown	1458	48.2	660	16.7
Diabetes status:				
No diabetes	2581	85.3	3165	80.0
Type 1 diabetes	183	6.0	182	4.6
Type 2 diabetes	263	8.7	608	15.4

Table A2.3. Binary patient characteristics at HF diagnosis in each cohort, GP-diagnosed

	2001/2 cohort			2011/12 cohort		
Factor level	N present	Frequency	Percent	N present	Frequency	Percent
HF symptoms up to diagnosis:						
Presence of: Any heart failure symptom	3027	1566	51.7	3955	2325	58.8
Presence of: Breathlessness/SOB/SOBE	3027	1208	39.9	3955	1935	48.9
Presence of: Fatigue	3027	362	12.0	3955	547	13.8
Presence of: Ankle swelling	3027	338	11.2	3955	542	13.7
First-symptom presence of: Breathlessness/SOB/SOBE	3027	1030	34.0	3955	1631	41.2
First-symptom presence of: Fatigue	3027	283	9.3	3955	386	9.8
First-symptom presence of: Ankle swelling	3027	259	8.6	3955	347	8.8
Physiological indicators:						
eGFR below 60mL/min	31	15	48.4	2513	932	37.1
Social vulnerability indicators:						
Living alone	3027	101	3.3	3955	206	5.2
Widowed or bereaved	3027	216	7.1	3955	229	5.8
Comorbidity components:						
Comorbidity: 1 Atrial fibrillation	3027	619	20.4	3955	1320	33.4
Comorbidity: 2 Arrhythmia other than atrial fibrillation	3027	259	8.6	3955	632	16.0
Comorbidity: 3 Diabetes	3027	445	14.7	3955	788	19.9
Comorbidity: 4 Hypertension	3027	1229	40.6	3955	2435	61.6
Comorbidity: 5 Renal diseases	3027	89	2.9	3955	994	25.1
Comorbidity: 6 Myocarditis	3027	38	1.3	3955	126	3.2
Comorbidity: 7 Acute myocardial infarction	3027	318	10.5	3955	511	12.9
Comorbidity: 8 Congenital heart disease	3027	7	0.2	3955	30	0.8
Comorbidity: 9 Coronary heart disease	3027	826	27.3	3955	1299	32.8
Comorbidity: 10 Chronic pulmonary disease	3027	539	17.8	3955	809	20.5

Comorbidity: 11 Stroke	3027	202	6.7	3955	284	7.2
Comorbidity: 12 Peripheral vascular disease	3027	221	7.3	3955	317	8.0
Baseline NHS contacts in previous year:						
CABG	3027	18	0.6	3955	44	1.1
PTCA	3027	21	0.7	3955	184	4.7
Pacemaker	3027	24	0.8	3955	81	2.0
ICD	3027	0	0.0	3955	7	0.2
Any hospital dialysis	3027	6	0.2	3955	15	0.4
Any hospital bed admission	3027	1194	39.4	3955	2011	50.8
Elective bed admission without HF primary diagnosis	3027	613	20.3	3955	1065	26.9
Emergency bed admission without HF primary diagnosis	3027	832	27.5	3955	1412	35.7
Emergency non-HF bed admission (one day only)	3027	71	2.3	3955	284	7.2
Emergency non-HF bed admission (at least one night)	3027	794	26.2	3955	1266	32.0
A&E visit ending in admission	3027	0	0.0	3955	1061	26.8
A&E visit not ending in admission	3027	0	0.0	3955	847	21.4
OPD appointment attended	3027	0	0.0	3955	2980	75.3
OPD appointment not attended	3027	0	0.0	3955	1185	30.0
OPD appointment for cardiology	3027	0	0.0	3955	1348	34.1
OPD appointment specialty	3027	0	0.0	3955	3025	76.5
4+ minute GP appointment	3027	2473	81.7	3955	3830	96.8
4+ minute practice nurse appointment	3027	1423	47.0	3955	3024	76.5
Home visit appointment	3027	1025	33.9	3955	944	23.9
Out of hours appointment	3027	384	12.7	3955	574	14.5
GP reported non-attendance	3027	113	3.7	3955	186	4.7
Practice nurse reported non-attendance	3027	53	1.8	3955	159	4.0
CPRD recorded A&E visit	3027	136	4.5	3955	915	23.1
CPRD recorded OPD appointment	3027	747	24.7	3955	2577	65.2
Heart failure review	3027	0	0.0	3955	2	0.1
Beta blockers (BNF Chapter 2.4)	3027	754	24.9	3955	1790	45.3

Thiazide-related diuretics (BNF Chapter 2.2.1)	3027	617	20.4	3955	731	18.5
Loop diuretics (BNF Chapter 2.2.2)	3027	1273	42.1	3955	1695	42.9
Aldosterone antagonists (spironolactone or eplerenone)	3027	87	2.9	3955	219	5.5
Renin-angiotensin system (RAS) drugs (BNF Chapter 2.5.5)	3027	1060	35.0	3955	2281	57.7
Glucocorticoid therapy (BNF Chapter 6.3.2)	3027	430	14.2	3955	695	17.6
Atypical antipsychotics (BNF Chapter 4.2.1.2 or drug names)	3027	26	0.9	3955	28	0.7

Table A3.1. Quantitative patient characteristics at time of first recorded HF diagnosis in each cohort, hospital-diagnosed

	2001/2 cohort			2011/12 cohort		
Quantitative variable	N present	Mean	SD	N present	Mean	SD
Attained age in diagnosis year	2954	76.9	11.4	8875	78.6	11.9
Number of comorbidities	2954	2.2	1.4	8875	3.2	1.6
Electronic frailty index (eFI)	2954	0.2	0.1	8875	0.2	0.1
BMI (kilos/square metre)	1359	27.2	5.6	6938	27.9	6.6
Systolic blood pressure (mm Hg)	2553	145.2	23.1	8690	133.7	19.5
Diastolic blood pressure (mm Hg)	2553	79.9	11.9	8690	74.4	11.5
Serum cholesterol (mmol/L)	1053	5.2	1.3	7505	4.6	1.2
HDL cholesterol (mmol/L)	491	1.3	0.4	6662	1.4	0.5
LDL cholesterol (mmol/L)	347	3.1	1.1	5736	2.5	1.0
HDL/LDL cholesterol ratio	215	4.1	1.6	5962	3.5	1.3
Blood glucose (mmol/L)	1156	7.6	4.2	6594	6.6	3.1
Serum creatinine (umol/L)	1663	110.6	46.4	8467	104.5	55.3
Serum triglycerides (mmol/L)	642	1.9	1.3	6190	1.5	0.9
Blood urea (mmol/L)	1168	8.0	5.2	7378	8.3	4.9
Estimated glomerular filtration rate (mL/min)	25	61.4	15.9	5492	60.3	21.0
Haemoglobin (g/dL)	1522	13.0	2.1	8201	12.7	2.0

Table A3.2. Categorical patient characteristics at HF diagnosis in each cohort, hospital-diagnosed

	2001/2 cohort		2011/12 cohort	
Factor level	Frequency	Percent	Frequency	Percent
All patients:				
Total	2954	100	8875	100
Patient Gender:				
Male	1454	49.2	4370	49.2
Female	1500	50.8	4505	50.8
Age group in diagnosis year:				
<45	36	1.2	94	1.1
45-64	371	12.6	1035	11.7
65-74	631	21.4	1566	17.6
75-84	1130	38.3	2964	33.4
85+	786	26.6	3216	36.2
IMD 2010 quintile:				
1 (least deprived)	458	15.5	1682	19.0
2	684	23.2	2081	23.4
3	582	19.7	1829	20.6
4	616	20.9	1787	20.1
5 (most deprived)	602	20.4	1489	16.8
Unknown	12	0.4	7	0.1
HES ethnicity (white or non-white):				
White	2546	86.2	8486	95.6
Non-white	49	1.7	290	3.3
Unknown	359	12.2	99	1.1
Number of comorbidities (group):				
0	255	8.6	257	2.9
1	704	23.8	1009	11.4
2	833	28.2	1964	22.1
3	634	21.5	2146	24.2
4+	528	17.9	3499	39.4
Electronic frailty index (eFI) category:				
Fit	1046	35.4	1327	15.0
Mild frailty	1416	47.9	3991	45.0
Moderate frailty	432	14.6	2929	33.0
Severe frailty	60	2.0	628	7.1
Smoking category (non, ex or current):				
Non-smoker	282	9.5	3511	39.6
Ex-smoker	189	6.4	3257	36.7
Current smoker	908	30.7	1492	16.8

Unknown	1575	53.3	615	6.9
Alcohol drinking group:				
Non drinker	123	4.2	1762	19.9
Light, moderate or unspecified	730	24.7	3057	34.4
Heavy or alcoholic	108	3.7	469	5.3
Unknown	1993	67.5	3587	40.4
BMI group:				
Underweight	46	1.6	265	3.0
Normal	442	15.0	2228	25.1
Overweight	508	17.2	2259	25.5
Obese	376	12.7	2218	25.0
Unknown	1582	53.6	1905	21.5
Diabetes status:				
No diabetes	2418	81.9	6640	74.8
Type 1 diabetes	195	6.6	550	6.2
Type 2 diabetes	341	11.5	1685	19.0

Table A3.3. Binary patient characteristics at HF diagnosis in each cohort, hospital-diagnosed

	2001/2 cohort			2011/12 cohort		
Factor level	N present	Frequency	Percent	N present	Frequency	Percent
HF symptoms up to diagnosis:						
Presence of: Any heart failure symptom	2954	1224	41.4	8875	4848	54.6
Presence of: Breathlessness/SOB/SOBE	2954	953	32.3	8875	3874	43.7
Presence of: Fatigue	2954	267	9.0	8875	1138	12.8
Presence of: Ankle swelling	2954	246	8.3	8875	1174	13.2
First-symptom presence of: Breathlessness/SOB/SOBE	2954	830	28.1	8875	3303	37.2
First-symptom presence of: Fatigue	2954	215	7.3	8875	814	9.2
First-symptom presence of: Ankle swelling	2954	181	6.1	8875	780	8.8
Physiological indicators:						
eGFR below 60mL/min	25	8	32.0	5492	2537	46.2
Social vulnerability indicators:						
Living alone	2954	241	8.2	8875	1013	11.4
Widowed or bereaved	2954	160	5.4	8875	532	6.0
Comorbidity components:						
Comorbidity: 1 Atrial fibrillation	2954	914	30.9	8875	4077	45.9
Comorbidity: 2 Arrhythmia other than atrial fibrillation	2954	358	12.1	8875	1714	19.3
Comorbidity: 3 Diabetes	2954	534	18.1	8875	2235	25.2
Comorbidity: 4 Hypertension	2954	1299	44.0	8875	6531	73.6
Comorbidity: 5 Renal diseases	2954	274	9.3	8875	3075	34.6
Comorbidity: 6 Myocarditis	2954	44	1.5	8875	203	2.3
Comorbidity: 7 Acute myocardial infarction	2954	631	21.4	8875	1718	19.4
Comorbidity: 8 Congenital heart disease	2954	13	0.4	8875	67	0.8
Comorbidity: 9 Coronary heart disease	2954	1231	41.7	8875	3820	43.0
Comorbidity: 10 Chronic pulmonary disease	2954	742	25.1	8875	2631	29.6

Comorbidity: 11 Stroke	2954	256	8.7	8875	856	9.6
Comorbidity: 12 Peripheral vascular disease	2954	296	10.0	8875	1165	13.1
Baseline NHS contacts in previous year:						
CABG	2954	19	0.6	8875	48	0.5
PTCA	2954	12	0.4	8875	130	1.5
Pacemaker	2954	28	0.9	8875	124	1.4
ICD	2954	0	0.0	8875	9	0.1
Any hospital dialysis	2954	9	0.3	8875	83	0.9
Any hospital bed admission	2954	1488	50.4	8875	5300	59.7
Elective bed admission without HF primary diagnosis	2954	701	23.7	8875	2502	28.2
Emergency bed admission without HF primary diagnosis	2954	1108	37.5	8875	4181	47.1
Emergency non-HF bed admission (one day only)	2954	66	2.2	8875	761	8.6
Emergency non-HF bed admission (at least one night)	2954	1079	36.5	8875	3907	44.0
A&E visit ending in admission	2954	0	0.0	8875	3962	44.6
A&E visit not ending in admission	2954	0	0.0	8875	2204	24.8
OPD appointment attended	2954	0	0.0	8875	6488	73.1
OPD appointment not attended	2954	0	0.0	8875	3168	35.7
OPD appointment for cardiology	2954	0	0.0	8875	2074	23.4
OPD appointment specialty	2954	0	0.0	8875	6680	75.3
4+ minute GP appointment	2954	2227	75.4	8875	8421	94.9
4+ minute practice nurse appointment	2954	1191	40.3	8875	6219	70.1
Home visit appointment	2954	1083	36.7	8875	3373	38.0
Out of hours appointment	2954	478	16.2	8875	1804	20.3
GP reported non-attendance	2954	108	3.7	8875	374	4.2
Practice nurse reported non-attendance	2954	57	1.9	8875	380	4.3
CPRD recorded A&E visit	2954	212	7.2	8875	3055	34.4
CPRD recorded OPD appointment	2954	757	25.6	8875	5651	63.7
Heart failure review	2954	0	0.0	8875	0	0.0

Beta blockers (BNF Chapter 2.4)	2954	699	23.7	8875	3223	36.3
Thiazide-related diuretics (BNF Chapter 2.2.1)	2954	541	18.3	8875	1703	19.2
Loop diuretics (BNF Chapter 2.2.2)	2954	1076	36.4	8875	3809	42.9
Aldosterone antagonists (spironolactone or eplerenone)	2954	99	3.4	8875	490	5.5
Renin-angiotensin system (RAS) drugs (BNF Chapter 2.5.5)	2954	843	28.5	8875	4686	52.8
Glucocorticoid therapy (BNF Chapter 6.3.2)	2954	440	14.9	8875	1769	19.9
Atypical antipsychotics (BNF Chapter 4.2.1.2 or drug names)	2954	33	1.1	8875	118	1.3
